# Multiscale Structural Design of 2D Nanomaterials‐based Flexible Electrodes for Wearable Energy Storage Applications

**DOI:** 10.1002/advs.202305558

**Published:** 2023-12-19

**Authors:** Yunfeng Chao, Yan Han, Zhiqi Chen, Dewei Chu, Qun Xu, Gordon Wallace, Caiyun Wang

**Affiliations:** ^1^ Henan Institute of Advanced Technology Zhengzhou University Zhengzhou 450052 China; ^2^ Intelligent Polymer Research Institute ARC Centre of Excellence for Electromaterials Science AIIM Facility Innovation Campus University of Wollongong Wollongong NSW 2522 Australia; ^3^ Energy & Materials Engineering Centre College of Physics and Materials Science Tianjin Normal University Tianjin 300387 China; ^4^ School of Materials Science and Engineering The University of New South Wales Sydney NSW 2052 Australia

**Keywords:** 2D Materials, energy storage devices, flexible electrodes, multiscale design strategies

## Abstract

2D nanomaterials play a critical role in realizing high‐performance flexible electrodes for wearable energy storge devices, owing to their merits of large surface area, high conductivity and high strength. The electrode is a complex system and the performance is determined by multiple and interrelated factors including the intrinsic properties of materials and the structures at different scales from macroscale to atomic scale. Multiscale design strategies have been developed to engineer the structures to exploit full potential and mitigate drawbacks of 2D materials. Analyzing the design strategies and understanding the working mechanisms are essential to facilitate the integration and harvest the synergistic effects. This review summarizes the multiscale design strategies from macroscale down to micro/nano‐scale structures and atomic‐scale structures for developing 2D nanomaterials‐based flexible electrodes. It starts with brief introduction of 2D nanomaterials, followed by analysis of structural design strategies at different scales focusing on the elucidation of structure‐property relationship, and ends with the presentation of challenges and future prospects. This review highlights the importance of integrating multiscale design strategies. Finding from this review may deepen the understanding of electrode performance and provide valuable guidelines for designing 2D nanomaterials‐based flexible electrodes.

## Graphical abstract

2D nanomaterials play a critical role in realizing high‐performance flexible electrodes for wearable energy storge devices. Multiscale design strategies have been developed to exploit full potential and mitigate drawbacks of 2D materials. Summarizing the multiscale design strategies from macroscale down to micro/nano‐scale structures and atomic‐scale structures can provide valuable guidelines for the future research.

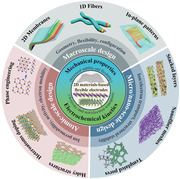

## 1 Introduction

The blooming of energy storage technology has been greatly revolutionizing our daily life to a more convenient and comfortable one than ever before. The wearable electronics such as earphones, smart watches/bands and glasses not only help to interact with the online world, but also provide intelligent assistance such as tracking and analyzing body data, and even saving life by enabling virtual care for remote patients.^[^ ^1^, ^2^, ^3^, ^4^ ^]^ The commonly used power sources to drive them are electrochemical energy storage (EES) devices, including rechargeable batteries and supercapacitors.^[^ ^5^ ^]^ Rechargeable batteries, also known as secondary batteries, store and deliver electrical energy through reversible chemical reactions with advantages of high energy density and long working life.^[^ ^5^, ^6^, ^7^ ^]^ Since the first commercialization of lithium‐ion batteries in 1990, the family of rechargeable batteries has been expanded to a series of metal‐ion batteries, metal‐sulfur batteries and metal‐air batteries (metals–Li, Na, K, Mg, Zn, etc.).^[^ ^8^, ^9^ ^]^ Supercapacitors, also called ultracapacitors, store electrical charge on the electrode‐electrolyte interface delivering electrochemical double layer capacitance (EDLC) or by Faradaic charge‐transfer via non‐diffusion limited redox reactions affording pseudocapacitance.^[^ ^10^, ^11^, ^12^ ^]^ The rapid kinetics and highly reversible processes enable excellent power performance and superlong lifetime. Energy storage devices for powering wearable electronics require excellent mechanical properties (high‐degree of flexibility and high strength), good electrochemical performance, and high safety. However, the conventional EES devices are rigid and bulky. When subjected to a deformation, the poor adhesion between the substrates and powdery active materials may lead to delamination causing high contact resistance, deteriorated electrochemical performance or even safety issues.^[^ ^13^, ^14^ ^]^ This calls for the improvement on the whole EES devices, especially the electrodes and electrolyte. As one of the key components that have a great impact on the flexibility, durability and performance, flexible electrode is becoming more and more important in the current research to develop advanced flexible EES devices.^[^ ^13^, ^15^ ^]^ The working mechanism in flexible electrodes follows the same fundamental principles for common electrodes: high ionic/electronic conductivity is necessary to facilitate fast electrochemical kinetics for excellent charge storage performance.^[^ ^16^ ^]^ In addition, flexible electrodes need to adapt to dynamic and deforming environments. Namely, they can deliver stable electrochemical performance under various deforming conditions,^[^ ^17^, ^18^ ^]^ which places requirements on the robustness and flexibility. It is essential to construct a robust structure with well‐connected conductive network and pathways for ensuring the fast ion/electron transport and the deformability.

2D nanomaterials have shown great potential for flexible electrodes due to their large surface area, good electrochemical activity, high conductivity and excellent mechanical properties.^[^ ^8^, ^19^, ^20^, ^21^ ^]^ They are a kind of nanomaterials in a sheet‐like layered structure with strong in‐plane covalent bonds and weak interlayer van der Waals interactions.^[^ ^19^ ^]^ They mainly include graphene‐family materials (e.g. graphene, graphene oxide), transition metal dichalcogenides (TMDs), MXenes, black phosphorus (BP), hexagonal boron nitride (h‐BN), and layered double hydroxides (LDHs).^[^ ^22^ ^]^ The weak van der Waals interaction between adjacent nanosheets is easy to break, facilitating the exfoliation of stacked layers to achieve large surface area.^[^ ^23^, ^24^ ^]^ The strong in‐plane covalent bonds endow 2D nanosheets with excellent mechanical properties.^[^ ^25^ ^]^ The high conductivity of 2D materials facilitates fast electron transfer for good rate performance. They can also be easily incorporated with other active materials to form high‐performance composites.^[^ ^24^, ^26^, ^27^ ^]^ Noteworthily, the large aspect ratio of 2D nanosheets enables the formation of liquid crystalline (LC) domains in the dispersion which greatly improves dispersibility and processability for liquid processing techniques.^[^ ^28^, ^29^, ^30^ ^]^ However, three main challenges are limiting their performance and practical applications: i) the restacking of 2D nanosheets due to π‐π stacking and van der Waals interactions severely limits the full use of large surface area;^[^ ^31^, ^32^ ^]^ ii) the close arrangement of atoms makes it impermeable for species larger than protons under ambient conditions, thus prolonging the diffusion pathways leading to deteriorative diffusivity and performance;^[^ ^33^, ^34^ ^]^ and iii) the volume change during the charging/discharging cycles causes structural damage which blocks diffusion pathways thus creating inaccessible active materials leading to degraded performance, or even safety issues when the separator is pierced as a result of significantly increased volume.^[^ ^35^ ^]^

The performance of flexible electrodes is determined by multiscale structures collectively influencing electrochemical reaction kinetics and structural stability. To maximize the potential of 2D nanomaterials for flexible electrodes, multiscale design strategies have been applied from macroscale to micro/nano scale and down to atomic scale.^[^ ^8^, ^36^ ^]^ Macroscale design focuses on improving the deformability of electrodes by engineering the geometry (i.e. 2D membranes, 1D fibers and in‐plane patterns), device configurations and connections. It aims to achieve good flexibility and mechanical robustness to meet the application scenarios.^[^ ^37^ ^]^ Micro/nano‐scale design is to engineer the morphology and porous structure of electrodes in order to promote electron/ion transport as well as address volume changes. Atomic‐scale design is developed to regulate the properties of 2D nanosheets via engineering atomic configurations.^[^ ^36^ ^]^ They are implemented to alter the intrinsic physicochemical properties such as conductivity, surface area, diffusivity and electrochemical activities. These multiscale design strategies are interrelated and complementary to each other. Intrinsic characteristics derived from atomic scale design are the fundamental determinant of electrochemical performance, as well as the processability of 2D nanosheets which plays a key role in implementing micro‐/nano‐ and macro‐scale design strategies. Micro/nano‐scale design strategies afford efficient utilization of the advantages of 2D materials, while macroscale design strategies realize the designed properties in the final application scenario.

**Figure 1:**
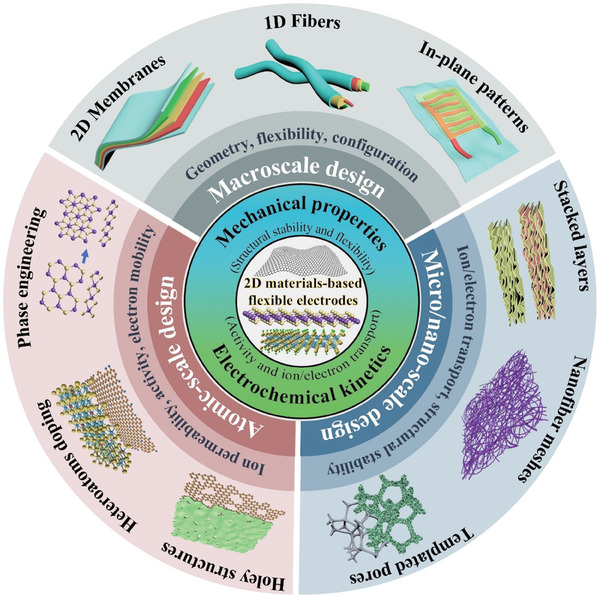
Multiscale structural design strategies for 2D materials‐based flexible electrodes.

There have been many excellent reviews about 2D nanomaterials^[^ ^38^, ^39^, ^40^, ^41^, ^42^, ^43^ ^]^ and flexible energy stoarge electrodes/devices^[^ ^3^, ^4^, ^44^, ^45^ ^]^. It is noted that reviews on 2D nanomaterials‐based flexible energy‐storage electrodes mainly focus on discussing the development from the aspects of electrode compositions^[^ ^25^, ^46^, ^47^ ^]^ or applications in different energy storage devices^[^ ^1^, ^48^, ^49^ ^]^. No review about the implementation of multiscale design strategies is available yet. In this review, we will systematically review the use of structural design strategies to engineer 2D materials‐based flexible electrodes. It will center on three commonly used 2D materials: graphene, TMDs and MXenes (**Figure** **1**). This review starts with a brief introduction of the fundamental properties and fabrication methods of 2D materials. Then it presents the implementation of structural design strategies from macroscale to atomic scale, which focuses on elucidating the strategies and structure‐property relationship. Finally, we will present current challenges and future opportunities facing the development of 2D materials‐based flexible electrodes.

## 2 2D Materials and Flexible Electrodes

### 2.1 2D Materials

The first member of 2D nanomaterials is graphene which was realized through a Scotch‐tape assisted exfoliation by Geim and Novoselo in 2004.^[^ ^19^ ^]^ Since then 2D materials have attracted great attention from academia and industry and found broad applications in various fields such as catalysis, energy storage as well as electromagnetic wave shielding and absorption.^[^ ^8^, ^36^ ^]^ Graphene, TMDs and MXenes are the most explored families for energy storage. Graphene is a kind of carbon material with covalently bonded carbon atoms in a honeycomb lattice structure,^[^ ^50^ ^]^ possessing a large specific surface area of 2630 m^2^ g^−1^, a high Young's modulus of up to 1 TPa, an intrinsic tensile strength of 130 GPa and a high electron mobility of 15 000 cm^2^ V^−1^ s^−1^.^[^ ^25^, ^50^, ^51^ ^]^ Transition metal dichalcogenides (TMDs) are typically in the form of MX_2_ with a plane of transition metal element (where M is Mo, W, Ti, etc.) sandwiched by two layers of chalcogen elements (X being S, Se, or Te).^[^ ^52^ ^]^ The high interlayer distance of TMDs is attractive for the insertion of large electrolyte ions. MXenes are a relatively new family of 2D materials discovered in 2011 by Barsoum and Gogotsi.^[^ ^53^, ^54^ ^]^ They are the collective name of 2D early transition metal carbides, nitrides and carbonitrides.^[^ ^55^, ^56^, ^57^, ^58^ ^]^ They are produced from MAX phases consisting of an early transition metal (M: Sc, Ti, Zr, Hf, V, Nb, Ta, Cr, Mo, W, or their combinations), a group IIIA or IVA element (A: Al, Ga, Si, or Ge) and carbon/nitrogen (X).^[^ ^59^, ^60^, ^61^ ^]^

**Figure 2:**
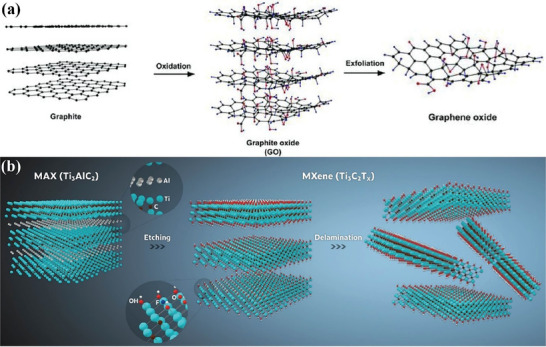
Schematic illustration of top‐down methods for producing exfoliated 2D nanosheets. a) Oxidation and exfoliation of graphite to form graphene oxide. Reproduced with permission.^[^ ^91^ ^]^ Copyright 2011, John Wiley and Sons. b) Etching synthesis of Ti_3_C_2_T_x_ MXene nanosheets. Reproduced with permission.^[^ ^81^ ^]^ Copyright 2020, Royal Society of Chemistry.

Tape‐assisted mechanical exfoliation can produce high‐quality graphene nanosheets, but with very low efficiency.^[^ ^62^ ^]^ Two commonly applied strategies to fabricate 2D materials are bottom‐up growing methods and top‐down exfoliating methods.^[^ ^25^ ^]^ Bottom‐up methods include chemical vapor deposition (CVD), electrodeposition and solvothermal synthesis,^[^ ^63^, ^64^ ^]^ where the growth of 2D nanosheets is regulated by substrates/templates into designed structures.^[^ ^65^ ^]^ As for top‐down strategies, mechanical and chemical approaches are the two main methods to produce 2D nanosheets. Mechanical exfoliation process is a typical top‐down approach using mechanical vibration energy to break the van der Waals attraction separating neighboring nanosheets.^[^ ^66^, ^67^ ^]^ Polar solvents such as N‐methyl‐2‐pyrrolidone (NMP)^[^ ^68^ ^]^ and surfactants (e.g. cetyltrimethylammonium bromide (CTAB),^[^ ^69^ ^]^ sodium dodecyl sulfate (SDS)^[^ ^66^, ^70^ ^]^) are commonly used to facilitate the exfoliation of 2D materials by matching the surface tension between solvents and molecules of 2D nanosheets.^[^ ^71^ ^]^ The van der Waals interactions between exfoliated nanosheets were also suppressed for an improved dispersibility. Chemical exfoliation methods based on ion/molecule‐intercalation strategies are attractive in terms of efficiency, scalability and controllability. Ions or molecules are inserted into the interlayers of bulk 2D materials to enlarge spacings and weaken interlayer interactions, thus facilitating exfoliation.^[^ ^72^, ^73^, ^74^ ^]^ The anchoring of surface groups on 2D nanosheets can produce highly stable dispersions with excellent processability.^[^ ^57^, ^59^ ^]^ For example, the modified Hummer's method uses potassium permanganate and concentrated sulfuric acid to expand bulk graphite (**Figure** **2**a).^[^ ^75^, ^76^ ^]^ The produced graphene oxide (GO) nanosheets possess rich oxygen‐containing functional groups (e.g. carboxyl, carbonyl, epoxy, hydroxyl, and lactol) on the surface and edges, generating electric repulsion between nanosheets for good dispersibility and processability.^[^ ^77^, ^78^ ^]^ The exfoliating of MAX to MXene nanosheets commonly involves an etching process with HF^[^ ^79^ ^]^ or molten salts^[^ ^80^ ^]^ to selectively remove element A in MAX phases. During the exfoliation process, different termination groups are generated on the surface producing terminated MXenes (M_n+1_X_n_T_x_) with good dispersibility and processability (Figure 2b).^[^ ^81^ ^]^ Those terminations can also be regulated to tune the properties of MXene nanosheets and improve intercalation pseudocapacitive behaviors.^[^ ^82^ ^]^

Stable dispersions are essential for multiscale design strategies using facile wet‐processing techniques.^[^ ^83^ ^]^ Zeta potential is an important indicator of the stability of colloidal dispersions. It directly relates to the surface charge groups or adsorbed ionic surfactant, which indicates the degree of electrostatic repulsion between adjacent nanosheets resisting aggregation.^[^ ^84^, ^85^ ^]^ Colloids with an absolute zeta potential value of above 30 mV are considered stable.^[^ ^70^ ^]^ As dispersion is a solid/liquid suspension system, the rheology property plays a key role in the processability.^[^ ^86^ ^]^ Rheology is mainly related to the aspect ratio and concentration of 2D nanosheets.^[^ ^83^ ^]^ Dispersions with low aspect ratio and/or low concentration of 2D nanosheets are viscoelastic liquids suitable for electrospray, ink‐jet printing, and casting/coating.^[^ ^87^ ^]^ Increasing the sheet concentration and lateral size can form liquid crystalline (LC) domains with parallelly orientated nanosheets. The excluded volume of GO nanosheets are compressed to minimize the orientational entropy and maximize the positional entropy, governing the formation of nematic LC phase.^[^ ^85^, ^88^, ^89^ ^]^ The LC dispersion functions as viscoelastic soft solid and fit for use in ink‐jet printing and wet‐spinning. LC dispersions of GO,^[^ ^86^, ^90^ ^]^ MoS_2_^[^ ^29^ ^]^ and MXene^[^ ^30^ ^]^ all have demonstrated robust LC domains. The dispersions with large aspect ratio and/or high concentration exhibit gel‐like behaviors with high elastic modulus, which are suitable for use in extrusion‐printing and even dry‐printing.^[^ ^83^ ^]^

### 2.2 Requirements of Flexible Electrodes

Energy storage devices for wearable electronics need to provide high energy and power densities as well as withstand mechanical deformations. Namely, they should have excellent flexibility to endure the deformation, possess good rate capability for quick charge and high energy density for long service time. As the key components for soft energy storage devices, flexible electrodes should possess high structural strength and fast ion/electron transport to meet the requirements. Structural strength serves as the foundation to achieve both flexibility and good structural stability. The high stress derived from deforming or volume expansion threatens the stable operation of flexible electrodes. On the other hand, high electronic conductivity and ion diffusivity allows for rapid reaction kinetics for achieving outstanding electrochemical performance. Considering that wearable electronics need quick charge for convenience, the promoting of ionic/electronic conductivity for an improved rate performance is of paramount importance for flexible electrodes.

The two main challenges facing the development of 2D materials‐based flexible electrodes are the volume expansion and low ionic conductivity. Volume changes are being occurred during the storing/releasing of electrolyte ions into/from the active materials, which can cause structural failure of flexible electrodes threating the stable operation and even safety of flexible energy storage devices. For example, the insertion of lithium ions into MoS_2_ exhibited a volume change of ≈103%^[^ ^92^ ^]^ while the formation of Li_3_P from BP during the alloying reaction is accompanied by a volume expansion up to 300%^[^ ^93^ ^]^. Such a huge volume change can result in structural failure for serious performance dropping and deteriorated deformability. Ion diffusivity is another challenge that resulted from the impermeable nature and restacking problem of 2D materials.^[^ ^32^, ^34^ ^]^ Impermeable 2D layers allow the ion transfer along their surface but prevent the ion diffusion through the basal planes. Restacking problem can reduce the surface area and interlayer distance to prolong the diffusion pathways. They can result in undesirable ion diffusivity to limit the charge storage performance.

Recently, different multiscale structures have been designed to effectively address these two challenges for developing high performance flexible electrodes. As research in this field continues to advance, the increasing types of multiscale structures make flexible electrodes more complex. Urgent attention is required to elucidate the development and functions of diverse structures providing valuable insights into understanding and predicting the future developments in the field of flexible electrodes.

## 3 Design Strategies for 2D Materials‐based Flexible Electrodes

To improve the performance of flexible electrodes, multiscale structures have been developed and designed to incorporate multiple length scales, ranging from macroscopic to atomic level. They interact with each other to form a complex system influencing electron/ion transport, electrochemical reaction process, as well as deformability. Understanding the design strategies and their influences on performance are required to implement the structural integration for developing flexible electrodes.

### 3.1 Macroscale Design Strategies

Macroscale structural design strategies are applied to realize deformability while sustaining electrochemical performance by engineering the electrodes geometry, device configurations and device connections. The deformation may include bending, folding, twisting and stretching while flexible electrodes are mainly in shapes of 2D membranes, 1D fibers and in‐plane patterns.

#### 3.1.1 2D Membranes

2D membrane electrodes, also known as paper or film electrodes, are bendable or even foldable. The assembled flexible devices have a sandwich configuration with two membrane electrodes and gel/solid‐state electrolyte in‐between. The commonly used fabrication methods include vacuum filtration, casting/coating, gelation, templating and roll‐pressing.

**Figure 3:**
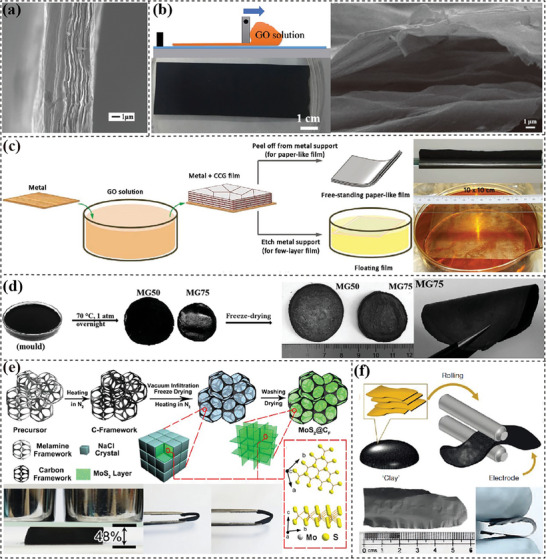
Fabrication of 2D materials‐based membrane electrodes. a) Filtered graphene paper as anodes in LIBs. Reproduced with permission.^[^ ^94^ ^]^ Copyright 2009, American Chemical Society. b) Chemical reduction of blade‐coated LCGO film for supercapacitors electrodes. Reproduced with permission.^[^ ^101^ ^]^ Copyright 2016, IOP Publishing. c) Schematic illustration of metal‐induced formation of rGO hydrogel films and the demonstration of rGO film formed on Cu. Reproduced with permission.^[^ ^106^ ^]^ Copyright 2013, John Wiley and Sons. d) MoS_2_‐rGO hydrogel film prepared from a low‐temperature hydrothermal gelation process. Reproduced with permission.^[^ ^113^ ^]^ Copyright 2017, John Wiley and Sons. e) Flexible MoS_2_/carbon foam electrode based on a melamine sponge substrate. Reproduced with permission.^[^ ^122^ ^]^ Copyright 2018, Elsevier. f) A rolling way to process the “clay” precursor into flexible MXene films. Reproduced with permission.^[^ ^128^ ^]^ Copyright 2014, Nature.

***Vacuum filtration*** is a simple and popular method to process liquid‐dispersed precursors into membrane electrodes. The filtration membrane allows the fast removal of solvents and holds back 2D nanosheets forming flexible films. The closely stacked nanosheets endow membrane electrodes with high strength and excellent flexibility. In 2009, our group^[^ ^94^ ^]^ first reported graphene film electrodes for lithium batteries by filtering reduced graphene aqueous dispersion (**Figure** **3**a). It exhibited a high capacity of 680 mAh g^−1^ at 0.05 A g^−1^ in the first cycle but dropped dramatically to 84 mA h g^−1^ at the second cycle due to the restacking issue. Alternatively, filtration of different dispersions and applying mixed precursors have been developed to form composite films, in order to overcome the stacking problem as well as provide additional energy storge contribution.^[^ ^95^, ^96^, ^97^ ^]^

***Casting/coating approaches*** include spin‐coating, blade‐coating, dip‐coating and spray‐coating.^[^ ^98^, ^99^ ^]^ The distance between 2D nanosheets decreases with the gradual removal of solvents, eventually forming flexible films.^[^ ^100^ ^]^ Wang and coworkers^[^ ^101^ ^]^ employed blade‐coating technique to process LCGO dispersion (Figure 3b). The formed rGO film after a reduction process displayed good flexibility and a high capacitance of 265 F g^−1^ (52 mF cm^−2^) in H_2_SO_4_ electrolyte. This strategy can also be used to directly produce flexible devices via sequentially coating anode, electrolyte and cathode. For example, the loading of anode material (rGO‐MoS_2_/N‐doped carbon) and cathode material (GO‐LiVPO_4_F/LiMn_2_O_4_) onto the opposite sides of a SiO_2_ modified PE separator, formed an integrated battery which displayed a stable capacity retention rate of 90% under a bending state.^[^ ^98^ ^]^ This strategy possesses the merits of simplicity, scalability and high efficiency, and has the potential to be combined with the industrialized roll‐to‐roll process that is applicable for mass production.

***Gelation*** is a widely used method to process dispersions into 3D hydrogels and then membrane electrodes.^[^ ^102^, ^103^ ^]^ The removal of surface groups on 2D nanosheets via reduction induces a self‐assembled gelation.^[^ ^104^, ^105^ ^]^ The 3D porous structures of hydrogels enable a good contact with electrolyte as well as effectively suppress volume change. In 2013, metal plates (Cu, Ni, Co, Fe and Zn) were used to induce the self‐assembly of GO nanosheets producing flexible rGO hydrogel films.^[^ ^106^ ^]^ This process relies on the oxidation‐reduction reaction occurred between metal and oxygen‐containing functional groups on GO. The used metals, dispersion concentration and gelation time were tuned to produce either floating few‐layer transparent films or freestanding paper‐like films (Figure 3c).^[^ ^106^ ^]^ The oxygen‐containing functional groups on MXene nanosheets can also be reduced by Zn to induce self‐assembly.^[^ ^104^, ^105^ ^]^ The capability of incorporating with other nanomaterials (e.g. Si and CNT) and the simplicity of controlling hydrogel shapes with Zn substrates were also demonstrated.^[^ ^107^ ^]^ High temperature and pressure are applied in a typical solvothermal gelation process.^[^ ^108^, ^109^, ^110^ ^]^ The hydrothermally formed hydrogels are commonly in column shape which need to be cut and compressed into thin and flexible membrane electrodes with improved strength and flexibility.^[^ ^110^, ^111^, ^112^ ^]^ Our group^[^ ^113^ ^]^ developed a low‐temperature hydrothermal process to directly prepare MoS_2_/rGO membrane (Figure 3d). The presence of LC state in the precursor dispersion facilitated the formation of orderly nematic liquid crystals which enabled the face‐to‐face contact with sufficient interactions between LCGO nanosheets and 1T‐rich MoS_2_ nanosheets, thus promoting the gelation process to form freestanding porous hydrogels.

***Templating method*** is used to guide the growth of 2D nanosheets. Carbon‐based substrates are typically used owing to their high corrosion resistance, good electrical conductivity, high strength and good bonding with many materials. They include carbon paper,^[^ ^114^ ^]^ carbon cloth,^[^ ^115^, ^116^, ^117^ ^]^ graphene foam,^[^ ^118^, ^119^, ^120^ ^]^ carbonized biomass materials,^[^ ^114^, ^121^ ^]^ and polymers^[^ ^122^, ^123^, ^124^ ^]^ Polymer‐derived carbon substrates have attracted attention due to their high processability facilitating the construction of various structures. Zhao's group^[^ ^122^ ^]^ used carbonized melamine sponge to load hierarchical MoS_2_ (Figure 3e). Flexible MoS_2_@carbon foam (MoS_2_@C_F_) electrode displayed excellent flexibility including compressibility, bendability, and recoverability. Moreover, these carbonaceous substrates are usually retained in the electrodes, and their conductive, flexible and robust nature facilitates the realization of high‐performance flexible electrodes.

***Roll‐pressing*** strategies are used to process mushy or solid precursors into freestanding and flexible films.^[^ ^125^, ^126^, ^127^ ^]^ Gogotsi's group^[^ ^128^ ^]^ processed “clay” MXene sediment into highly conductive and flexible MXene films (Figure 3f). Upon wetting, these films swelled to afford expanded interlayer spacing and showed thickness‐dependent capacitances. In Yu's work,^[^ ^126^ ^]^ graphite on lithium plate was repeatedly pressed to fabricate flexible lithium/graphene anodes. The resultant defect‐free and shape‐adaptive graphene nanosheets well protected micro‐sized Li metal particles from pulverization and limited the growth of Li dendrites. The roll‐pressing strategy is also shown as an emerging technique for fabricating dry battery electrodes, which is highly desirable in the battery industry to avoid the use of toxic organic solvents, simplify the manufacturing process, reconstruct electrode microstructures as well as improve the electrode‐electrolyte interface compatibility.^[^ ^129^, ^130^ ^]^

In addition, the thickness of membrane electrodes is also an important aspect of macroscale structures affecting the charge storage performance and flexibility. High charge storage performance is expected from the increased amount of active materials associated with the increased thickness. However, large thickness also presents challenges, such as sluggish reaction kinetics owing to the prolonged pathways of ion/electron and reduced flexibility due to the increased stress during deforming. For example, MoS_2_/graphene aerogel films demonstrated the highest areal capacity at a thickness of 173 𝜇m (8.2 mg cm^−2^), and the capacity dropped in larger thickness accompanied by the occurrence of cracks during the bending.^[^ ^28^ ^]^ Free‐standing MoS_2_/SWNT membranes exhibited a thickness‐dependent electrochemical behavior before reaching the best performance in the range between 52–72 𝜇m.^[^ ^131^ ^]^ Therefore, the thickness of membrane needs to be optimized to achieve the best performance.

#### 3.1.2 1D Fibers

1D fiber electrodes can be arranged in parallel,^[^ ^132^ ^]^ twisted^[^ ^133^ ^]^ or concentrical^[^ ^134^ ^]^ configurations in fiber‐shape energy storage devices with omnidirectional flexibility. They are particularly attractive for being woven into wearable textile‐type arrays or integrating with commercial fabrics.^[^ ^135^, ^136^, ^137^ ^]^ Moreover, they can be coiled into a spring structure to acquire superior stretchability and broaden the application ranges.^[^ ^46^ ^]^ 1D fiber electrodes are mainly produced via templating, wet‐spinning, and microfluidic spinning.

**Figure 4:**
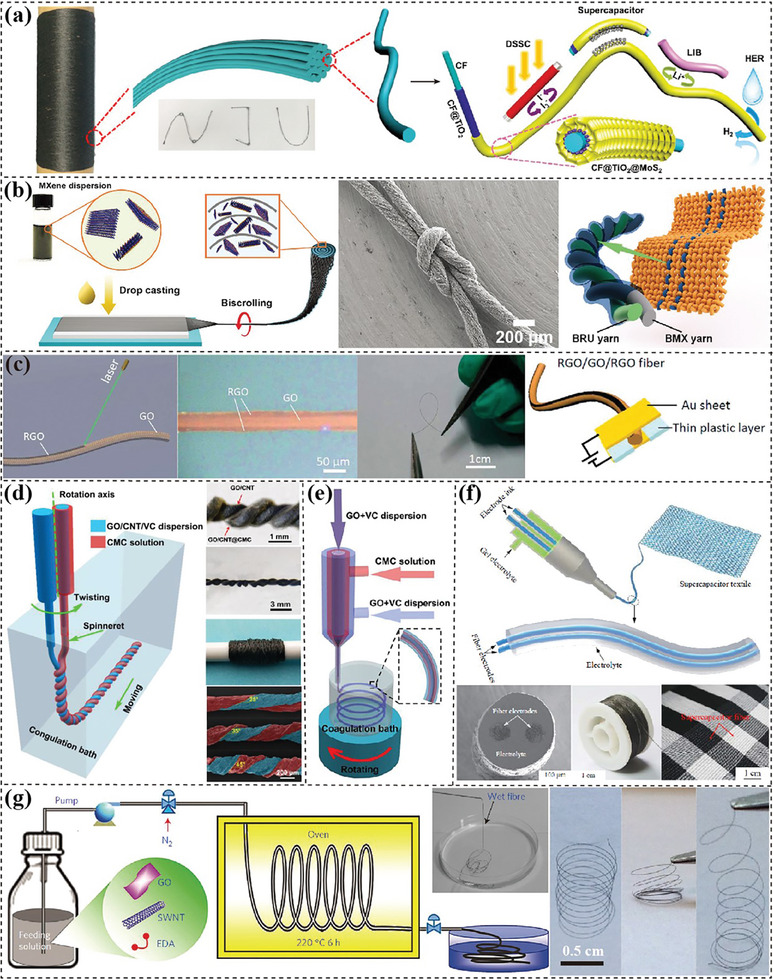
Fabrication of fiber electrodes. a) Coaxial fibrous CF@TiO_2_@MoS_2_ electrodes for energy harvesting and storage. Reproduced with permission.^[^ ^141^ ^]^ Copyright 2016, John Wiley and Sons. b) Biscrolled CNT substrates and drop‐casted MXene for flexible BMX yarn and the demonstration of twisted asymmetric FSCs with biscrolled CNT/RuO_2_ yarn as counter electrodes. Reproduced with permission.^[^ ^142^ ^]^ Copyright 2015, John Wiley and Sons. c) All‐in‐one RGO/GO/RGO FSC prepared from a wet‐spun GO fiber. Reproduced with permission.^[^ ^148^ ^]^ Copyright 2014, Royal Society of Chemistry. d) Wet‐spinning of twisted fiber‐shape supercapacitors. Reproduced with permission,^[^ ^133^ ^]^ Copyright 2020, Elsevier. e) Wet‐spinning of FSCs using a concentric apparatus. Reproduced with permission.^[^ ^134^ ^]^ Copyright 2019, Elsevier. f) Wet‐spinning of FSCs with parallel electrodes and its application demonstration. Reproduced with permission.^[^ ^132^ ^]^ Copyright 2019, Springer. g) Hydrothermally synthesized rGO/CNT fibers displaying excellent flexibility. Reproduced with permission.^[^ ^156^ ^]^ Copyright 2014, Nature.

***Templating methods*** use wire‐ or fiber‐shape substrates as templates to guide the growth of active materials forming fiber electrodes.^[^ ^138^, ^139^, ^140^ ^]^ In Liang's work,^[^ ^141^ ^]^ commercially available carbon fibers (CFs) were employed for preparing coaxial fibrous CF@TiO_2_@MoS_2_ electrodes (**Figure** **4**a). The good contact of active materials with CF enables excellent performance in versatile fiber‐shaped flexible energy harvesting and storage devices including LIBs and supercapacitors. CNT forest is another commonly used substrate for loading active materials and twisting into fibrous yarns.^[^ ^142^, ^143^ ^]^ Razal's group^[^ ^136^ ^]^ drop‐casted MXene dispersion onto CNT stacked mats and then twisted them into yarns (Figure 4b). Even with a high MXene content of ≈98 wt.%, MXene/CNTs yarns still demonstrated remarkable flexibility, mechanical durability, and excellent capacitance performance.

***Wet‐spinning*** is a simple and scalable method to prepare fiber‐shaped electrodes by continuously injecting ink precursor into a coagulation bath.^[^ ^30^, ^144^, ^145^ ^]^ The rheological properties required for wet‐spinning can be acquired by manipulating the dispersion.^[^ ^29^, ^83^, ^146^ ^]^ When the dispersion flows through the needle during injection, a shear‐induced deformation arranges the randomly dispersed nanosheets into an orderly aligned structure.^[^ ^30^, ^146^ ^]^ After being injected into coagulation bath, the solvent‐exchanging process induced the self‐assembly of 2D nanosheets forming robust 1D hydrogel fibers.^[^ ^29^, ^30^, ^86^ ^]^ In 2014, Gao's group developed a continuous wet‐spun belt by utilizing LCGO dispersion.^[^ ^147^ ^]^ Hu and coworkers^[^ ^148^ ^]^ employed laser irradiation to selectively reduce wet‐spun GO fiber for fabricating all‐in‐one fiber supercapacitor with untreated part acting as separator (Figure 4c). The rGO–GO–rGO fiber maintained a high tensile strength of 150 MPa and great flexibility. Multi‐channels spinnerets can concurrently spin different materials for fabricating hollow‐structured electrodes, electrolyte‐coated electrodes and even whole energy storage devices.^[^ ^149^, ^150^ ^]^ Our group^[^ ^151^ ^]^ employed reductant‐containing coagulant solution as core to produce hollow graphene fibers with improved conductivity and large contact area with electrolyte. Both the outer layer and inner layer were accessed by electrolyte and delivered a high capacitance of 711 mF cm^−2^ (33.5 mF cm^−1^ or 190 F cm^−3^) in PVA/H3PO_4_ electrolyte. Yang and coworkers^[^ ^133^ ^]^ used a coaxial wet‐spinning apparatus to spin rGO/CNT fibers with electrolyte as shell, then twisted together forming a fiber supercapacitor in the coagulation bath (Figure 4d), which eliminated an electrolyte‐coating procedure. One‐step spinning of whole fiber‐shaped devices is realized via using a specially designed syringe. Li et al.^[^ ^134^ ^]^ utilized a triaxial syringe to directly prepare fiber devices in concentric configuration (Figure 4e). The thin middle layer of gel electrolyte was merged with the core and sheath to form wrinkled interfaces with enhanced contact area between active materials. In Yang's work,^[^ ^132^ ^]^ a multichannel spinneret was elaborately designed with two parallel inner nozzles for electrode inks and one outer larger nozzle carrying gel electrolyte, to continuously fabricate parallel FSCs (Figure 4f). The production rate reached as high as 118 m h^−1^ with well‐maintained flexibility and electrochemical properties. These robust fibers were woven together with cotton fibers into a large‐scale scarf.

***Microfluidic spinning*** is a simple method for preparing fibrous electrodes through hydrothermal self‐assembly processes confined in a thin and long container.^[^ ^152^, ^153^, ^154^ ^]^ GO‐based dispersions are usually used due to their good mechanical properties and facile hydrothermal gelation process. Early in 2012, rGO hydrogel fibers with high strength and low density were obtained by heating the GO dispersion in a glass pipeline.^[^ ^155^ ^]^ Chen's group^[^ ^156^ ^]^ further developed a scalable method to continuously produce hierarchically structured CNT‐graphene fiber by pumping the precursor into a fused silica capillary column in an oven at 220 °C (Figure 4g). The resultant fibers showed excellent flexibility and high stretchability after being coiled into a spring‐shape.

Similar as the effect from membrane thicknesses, the diameter of fibrous electrodes also affects their fracture strength, flexibility and electrochemical performance. Generally, the increased diameter can result in decreased tension strength and thus reduced flexibility.^[^ ^157^ ^]^ The linear capacity/capacitance is increased with the fiber diameter before reaching a upper limit, and the ionic conductivity becomes worsened for fibers in even higher diameter along with decreased utilization rate of active materials.^[^ ^158^ ^]^ In Zhang's work,^[^ ^159^ ^]^ the linear capacities of graphene/CNT/SnO_2_ fibers increased as the increase in diameter, but decreased when the diameter surpassed 880 𝜇m. An optimal diameter is necessary to ensure a high utilization rate of active materials and achieve the best overall performance.

#### 3.1.3 In‐Plane Patterns

In‐plane patterned electrodes have attracted great interest for fabricating micro‐sized EES devices to power microscale electronics or to constitute integrated arrays.^[^ ^160^ ^]^ The in‐plane microdevices achieve flexibility by itself,^[^ ^160^, ^161^, ^162^, ^163^ ^]^ or through soft bridging structures.^[^ ^164^, ^165^ ^]^ These patterned electrodes are commonly shaped into interdigitated structures with periodic finger electrodes. Generally, in‐plane patterns are fabricated on substrates through printing or etching methods. The fabrication strategies can be classified into three types: pattern‐shaping, additive manufacturing and laser reduction/etching.

**Figure 5:**
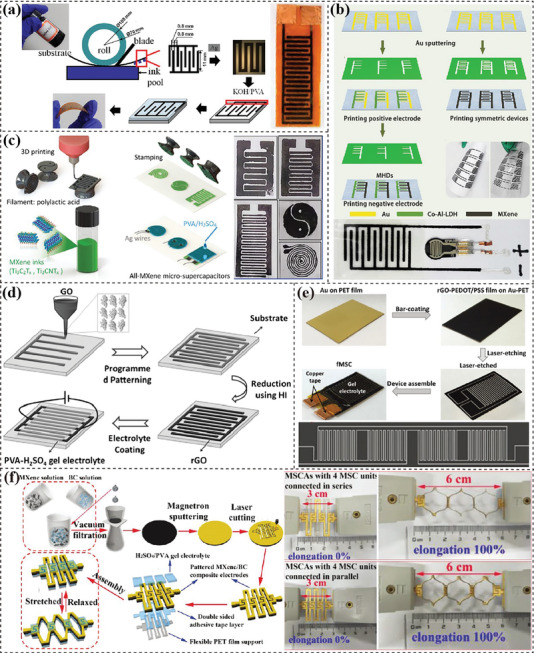
Fabrication of in‐plane patterned electrodes. a) Gravure printing of hybrid MoS_2_@S‐rGO interdigitated electrodes for MSCs. Reproduced with permission.^[^ ^167^ ^]^ Copyright 2015, AIP Publishing. b) Asymmetric MSC screen‐printed from MXene and Co‐Al layered double hydroxide inks and its application powering a force sensing resistor. Reproduced with permission.^[^ ^170^ ^]^ Copyright 2018, Elsevier. c) Stamp‐printing of flexible and planar MXene MSCs. Reproduced with permission.^[^ ^162^ ^]^ Copyright 2018, John Wiley and Sons. d) Extrusion‐printed interdigital GO layers for planar MSCs. Reproduced with permission.^[^ ^180^ ^]^ Copyright 2015, Elsevier. f) Laser etching of MXene/bacterial cellulose paper to form interdigital MSCs in a stretchable Kirigami configuration. Reproduced with permission.^[^ ^190^ ^]^ Copyright 2019, John Wiley and Sons.

***Pattern‐shaping strategy*** employs different patterns to regulate precursor inks into desired in‐plane electrodes, which mainly includes gravure printing, screen printing and stamp‐printing. Gravure printing is a traditional roll‐to‐roll technique using a gravure roller or plate to load inks in grooves and then deposit on the substrate with the assistance of a doctor blade to remove excess inks.^[^ ^166^ ^]^ Huang et al.^[^ ^167^ ^]^ employed this technique to print a mixed ink of MoS_2_ and sulfonated rGO into interdigitated electrodes on flexible polyimide (**Figure** **5**a). The produced device displayed good flexibility and electrochemical performance. Screen printing employs patterned mold as mask to process inks into different patterns.^[^ ^168^, ^169^ ^]^ Han and coworkers^[^ ^170^ ^]^ used interdigital molds to print asymmetric microsupercapacitors (MSCs) through a successive screen‐printing of Ti_3_C_2_T_x_ and Co‐Al layered double hydroxide (Co‐Al‐LDH) inks (Figure 5b). Normally customized molds are designed to prepare in‐plane electrodes.^[^ ^171^, ^172^ ^]^ Nicolosi's group^[^ ^162^ ^]^ applied a 3D printing technique to prepare patterned stamps including interdigitated, Yin Yang and spiral shapes (Figure 5c) for constructing in‐plane supercapacitors. The cylindrical stamp could even be rolled for rapid fabrication of devices, demonstrating the potential for scale‐up production.

***Additive manufacturing*** is adopted to prepare patterned electrodes by continuously loading layer upon layer of precursor inks onto substrates through a spinneret. This strategy holds the potential of in‐situ fabricating whole devices through sequentially printing electrodes and electrolyte. Inkjet printing is an additive manufacturing method that allows the use of low‐concentration inks to process ink droplets into patterns with precise control and high speed.^[^ ^173^, ^174^, ^175^, ^176^ ^]^ In Shao's work,^[^ ^177^ ^]^ inkjet‐printing was applied to prepare flexible asymmetric MSCs with rGO and 1T‐MoS_2_ as electrodes. The MoS_2_ electrodes with a high content of 1T phase enlarged the potential range to 1.75 V in a bivalent magnesium‐ion aqueous electrolyte, and displayed an improved performance with a 96% capacitance retention rate over 20 000 cycles. Compared with inkjet printing, extrusion printing employs high‐concentration inks for fabricating 3D patterns layer‐by‐layer.^[^ ^178^, ^179^ ^]^ Inks for this technique require a shear thinning behavior for good flowability during the printing process and high viscosity at low shear rate to hold the printed structure.^[^ ^163^ ^]^ Sun et al.^[^ ^180^ ^]^ used a GO dispersion of 20 mg ml^−1^ as ink for extrusion printing interdigital electrodes with multilayer patterns (Figure 5d), which displayed a linearly increased areal performance with thickness (i.e. layers).

***Laser reduction/etching*** is conducted to process prefabricated membranes into in‐plane patterns through two main strategies: laser reduction and laser etching. Compared with other patterning methods, they are not only time efficient but also accurate in constructing in‐plane microsized electrodes owing to the good stability and easy controllability of laser.^[^ ^181^ ^]^ The laser reduction method was commonly applied on GO films to prepare interdigitated rGO electrodes, in which a low‐energy laser removed the surface functional groups on GO nanosheets without damaging the basal planes. For example, Maher and coworkers^[^ ^182^ ^]^ used laser from a commercial DVD burner to process thin GO layer into “laser‐scribed graphene (LSG)” patterns with a lateral spatial resolution of ≈20 µm. A single laser scanning can produce more than 100 planar MSCs, delivering a high capacitance of 3.05 F cm^−3^ at 16.8 mA cm^−3^ with only 4% drop after 10 000 cycles. The laser etching way was applied to engrave the substrates into designed patterns, in which the contact area with high‐energy laser was etched out.^[^ ^183^, ^184^, ^185^ ^]^ Chen's group^[^ ^186^ ^]^ used laser to engrave out interdigitated patterns on rGO‐PEDOT/PSS film on Au‐coated PET (Figure 5e). They demonstrated the high efficiency of this strategy by fabricating ten different MSCs devices in a 100‐second laser etching run. Moreover, laser etching was also applied to design structure bridging patterned microdevices. Excellent stretchability was achieved by creating self‐similar serpentine interconnection,^[^ ^187^ ^]^ island‐bridge network^[^ ^188^ ^]^ and kirigami configuration.^[^ ^189^ ^]^ Jiao et al.^[^ ^190^ ^]^ sputtered a gold layer on filtered MXene/bacterial cellulose composite film to fabricate MSC arrays via laser‐etching (Figure 5f). Micro‐sized electrodes and connection bridges were arranged in kirigami configuration and showed a high elongation ratio of 100% in both series and parallel connections.

### 3.2 Micro/Nano‐Scale Design Strategies

Micro‐nano scale design strategies focus on engineering the morphology and 3D porous structure of electrode material, which play a critical role in enhancing mechanical strength and ion/electron transport. 2D nanosheets with large aspect ratio and high mechanical strength can be easily assembled into different nanostructures and achieve high surface area for improved contact with electrolyte, high porosity for accommodating volume expansion, interconnected channels for fast ion diffusion and 3D conductive network for rapid electron transfer. They mainly include nanoparticles, nanowires/nanofibers, nanotubes, hollow structures, layered structures, and hierarchical structures. These nanofeatures mainly form three types of structures in flexible electrodes: stacked layers, nanofiber meshes and templated pores.^[^ ^49^, ^191^, ^192^ ^]^

#### 3.2.1 Stacked Layers

The π‐π stacking and van der Waals interactions between 2D nanosheets facilitate the stacking into layered arrangement through wet‐processing methods such as vacuum filtration,^[^ ^193^, ^194^ ^]^ casting/coating methods^[^ ^195^ ^]^ and wet‐spinning.^[^ ^29^ ^]^ The sub‐nanometer channels are engineered to promote ion diffusivity by taking advantage of nanoconfinement effects.^[^ ^196^ ^]^ However, the close contact between stacked layers always causes a restacking problem, limiting interlayer spacing and surface area.^[^ ^94^, ^197^, ^198^ ^]^ The incorporation with other active materials to form composites in a sandwich structure is a common solution via layer‐by‐layer processing, directly mixing and surface decorating.

**Figure 6:**
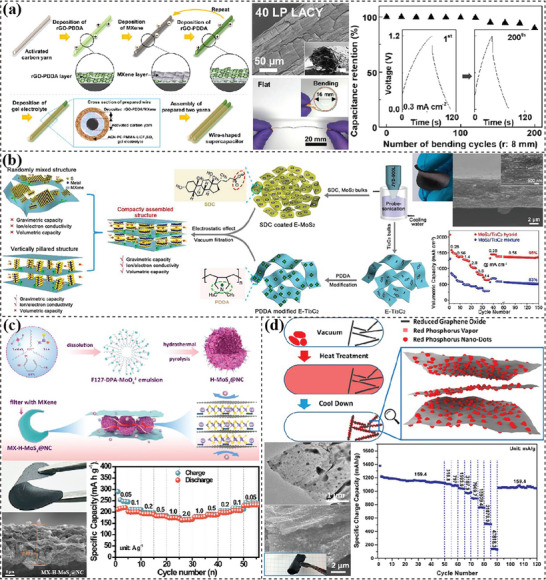
2D materials‐based flexible electrodes with sheet‐stacked layer structures. a) LBL assembly of rGO and MXene nanosheets on active carbon yarns. Reproduced with permission.^[^ ^202^ ^]^ Copyright 2021, American Chemical Society. b) Mixing stacked layers with vertically pillared MoS_2_ nanosheets sandwiched between horizontal MXene nanosheets. Reproduced with permission.^[^ ^207^ ^]^ Copyright 2020, Elsevier. c) Flexible layered film filtrated from hollow MoS_2_/nitrogen‐doped carbon spheres and MXene nanosheets. Reproduced with permission.^[^ ^211^ ^]^ Copyright 2022, Elsevier. d) Infiltration of red phosphorus onto rGO nanosheets. Reproduced with permission.^[^ ^213^ ^]^ Copyright 2017, American Chemical Society.

Layer‐by‐layer (LBL) strategy refers to the alternately processing of two or two different materials repeatedly via vacuum filtration,^[^ ^96^, ^97^ ^]^ spray coating,^[^ ^199^, ^200^ ^]^ dip‐coating^[^ ^201^, ^202^ ^]^ and spin‐coating.^[^ ^98^, ^203^ ^]^ Gogotsi's group^[^ ^199^ ^]^ reported an alternately spray‐coating process to make Ti_3_C_2_T_x_/graphene paper. The face‐to‐face contact between Ti_3_C_2_T_x_ and graphene facilitated the formation of van der Waals heterostructure and achieved a high sodium storage performance of 600 mAh g^−1^ at 0.25 C. Yun et al.^[^ ^202^ ^]^ prepared fibrous electrodes by repeatedly dipping active carbon yarns (ACY) in positively charged rGO dispersion functionalized with poly(diallyldimethylammonium chloride) (rGO‐PDDA) and negatively charged Ti_3_C_2_T_x_ MXene dispersion (**Figure** **6**a). A concentric layered structure with up to 40‐layer pairs was formed, and yielded a high capacitance of 237 F g^–1^ or 2193 F cm^–3^ with a high retention ratio of 90% after 200 bending tests at a bending radius of 8 mm.

Through stirring,^[^ ^204^ ^]^ ball‐milling^[^ ^205^ ^]^ and ultrasonication,^[^ ^206^ ^]^ other materials can be directly mixed with 2D nanosheets. Vacuum filtration is the most widely used method to process the mixture into flexible membrane electrodes with sandwich layers.^[^ ^193^ ^]^ The combination of two different 2D materials could effectively prevent the restacking and enhance ion diffusion as well as construct heterostructures maximizing charge storage. Utilizing the electrostatic effect between sodium deoxycholate‐functioned MoS_2_ and poly(diallyldimethylammonium chloride) modified Ti_3_C_2_ nanosheets, Li's groups^[^ ^207^ ^]^ intercalated parallel MoS_2_ nanosheets into horizontal MXene nanosheets forming covalently bonded heterostructures (Figure 6b). Stabilized by the 2D confinement effect and the Ti‐S‐Mo interfacial bonds, this robust and flexible hybrid film achieved a balanced porosity with a BET surface area of 16.4 m^2^ g^−1^ and a high density of ≈2.9 g cm^−3^. This contributed to an exceptional electrochemcial performance of 650 mAh cm^−3^ at 14 mA cm^−2^ and a linear relationship between areal capacity and film thickness. In addition to 2D nanosheets, other nanostructures including 1D nanofibers,^[^ ^69^, ^208^ ^]^ nanoparticles,^[^ ^194^, ^209^ ^]^ hierarchical structures^[^ ^210^ ^]^ and hollow structures^[^ ^135^, ^211^ ^]^ are also introduced to prepare sandwiched layers with self‐created micro/nanoscale channels. Hollow MoS_2_/nitrogen‐doped carbon (H‐MoS_2_@NC) spheres were synthesized first and then mixed with MXene nanosheets to be filtered into composite film (Figure 6c).^[^ ^211^ ^]^ Benefitting from the combination of hierarchical surface, hollow structure and sandwiched layers, a high‐speed interconnected network was constructed which greatly improved ion diffusion and thus reaction kinetics. The volume expansion problem was also alleviated, achieving a high capacity of 220 mAh g^−1^ at 0.05 A g^−1^, 170 mAh g^−1^ at 2 A g^−1^, and 182.3 mAh g^−1^ after 135 cycles at 0.2 A g^−1^ in full sodium ion batteries when coupled with Na3V_2_(PO_4_)_2_F_3_/C cathodes.

Different from the above mixing strategies, surface‐decorated layers are realized by depositing other materials on the surface of layered 2D nanosheets. They are applied for achieving fast electron transport and improved structure stability on account of the intimate contact.^[^ ^163^, ^212^ ^]^ The widely used loading approaches include vapor deposition,^[^ ^213^ ^]^ hydrothermal reaction,^[^ ^214^, ^215^ ^]^ polymerization^[^ ^163^ ^]^ and electrodeposition.^[^ ^216^, ^217^ ^]^ Vapor deposition is commonly used to deposit uniform and conformal layers on substrates via physical or chemical routes. Zhou's group^[^ ^213^ ^]^ employed this method to uniformly decorate red phosphorous onto rGO nanosheets (Figure 6d). The flexible P@RGO film displayed a sodium storage performance of 510.6 mAh g^−1^ at 31.8 A g^−1^. Wet‐chemical growing methods can achieve better interfacial bonding by utilizing the interactions between surface groups and ion precursors.^[^ ^214^ ^]^ In Liu's work,^[^ ^163^ ^]^ polyaniline (PANi) nanorods were vertically polymerized onto GO nanosheets through the interaction between positively charged aniline and negatively‐charged surface groups on GO nanosheets. With the assistant of PEDOT:PSS, the obtained GO/PANi composite was capable of dispersing well in water. Interdigitated electrodes with layered structure were then extrusion‐printed, which displayed excellent flexibility and a high capacitance of 153.6 mF cm^−2^ (19.2 F cm^−3^) at 5 mV s^−1^ in a symmetric all‐solid‐state supercapacitor. Besides, surface‐decorating layers can also be grown onto conductive films via electrodeposition.^[^ ^218^ ^]^ Our group^[^ ^218^ ^]^ decorated nodule‐like polypyrrole (PPy) onto rGO nanosheets of filtered wet rGO films via electrochemical polymerization. The areal capacity of hybrid rGO‐PPy film increased with the deposition time before reaching to a maximum 440 mF cm^−2^ at 0.5 A g^−1^ after 60‐min deposition. Tian et al.^[^ ^219^ ^]^ employed filtered MXene Ti_3_C_2_T_x_ film to electrochemically grow hierarchical flower‐like Sb particles. However, the restacked layers may have restricted the permeation of precursors and resulted in low and non‐uniform loading.^[^ ^220^ ^]^

#### 3.2.2 Nanofiber Meshes

**Figure 7:**
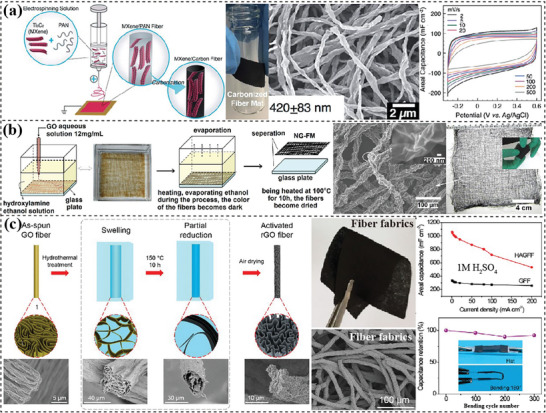
2D materials‐based flexible electrodes with interlaced fibers. a) Electrospun and annealed Ti_3_C_2_T_x_/carbon fiber film. Reproduced with permission.^[^ ^222^ ^]^ Copyright 2019, Royal Society of Chemistry. b) Wet‐spun and thermally reduced graphene fiber mats. Reproduced with permission.^[^ ^223^ ^]^ Copyright 2014, Elsevier. c) Wet‐spun, filtered and hydrothermally activated rGO fiber mats. Reproduced with permission.^[^ ^227^ ^]^ Copyright 2017, American Chemical Society.

The intertwining of nanofibers forms meshes with good flexibility and numerous channels for electrolyte access.^[^ ^221^ ^]^ Electrospinning, wet‐spinning, and microfluidic‐spinning are three commonly used approaches, during which nanofibers are first spun and then interlaced into an interconnected network. Electrospinning is a traditional method to process precursors into nanofibers being intertwined into mesh structures. Gogotsi's group^[^ ^222^ ^]^ fabricated flexible MXene/carbon fiber films by electrospinning the colloidal solution of polyacrylonitrile (PAN) and Ti_3_C_2_T_x_ MXene, followed by an annealing process (**Figure** **7**a). The diameter of fibers was decreased after carbonization while their integrity and flexibility were well retained. In a three‐electrode configuration, it delivered an areal capacitance of 239 mF cm^−2^ at 10 mV s^−1^. Wet‐spinning techniques were also altered to prepare interlaced fiber mats.^[^ ^223^, ^224^, ^225^, ^226^ ^]^ Chang and coworkers^[^ ^223^ ^]^ programmed the wet‐spinning process to construct a GO fiber mat structure (Figure 7b), which was then thermally reduced to form graphene fibers mats (GFM). The GFM annealed at 300 °C possessed a low density of 0.4 g cm^−3^, a large BET surface area of 170 m^2^ g^−1^ and a high electrical conductivity of 21 S cm^−1^. The assembled symmetric supercapacitor delivered a high capacity of 188 F g^−1^ at 5 mV s^−1^ with a capacity retention ratio of 48.4% at 10 V s^−1^ in a 25% KOH solution. Gao's group^[^ ^224^ ^]^ employed a simple filtration method to process microfluidic‐spun fibers into flexible fabrics. The wet‐spun GO fibers were fused together at the fiber junction, achieving excellent conductivity of 138.9 S m^−1^ and high tensile strength of 29.9 MPa after thermal reduction.^[^ ^247^ ^]^ After a further hydrothermal activation treatment, the rGO fiber meshes displayed a rough surface with a high surface area of 245 m^2^ g^−1^ (Figure 7c).^[^ ^227^ ^]^ As a result, these hydrothermally activated graphene fiber fabrics (HAGFFs) delivered a greatly improved capacitance of 1060 mF cm^−2^ at 1 mA cm^−2^ in 1M H_2_SO_4_ electrolyte compared with only 344 mF cm^−2^ for GFFs.

#### 3.2.3 Templated Pores

The stacking of 2D nanosheets forms horizontal interlayer channels and their incorporation with other nanostructured materials endows numerous pores and channels. To further increase the surface area, improve ion diffusivity and the ability to endure huge volume expansion, various templates are introduced to create controllable pores and interconnected channels.^[^ ^228^ ^]^ They include self‐generated/introduced gases,^[^ ^229^ ^]^ liquid droplets^[^ ^230^ ^]^ and solid templates.^[^ ^216^ ^]^

**Figure 8:**
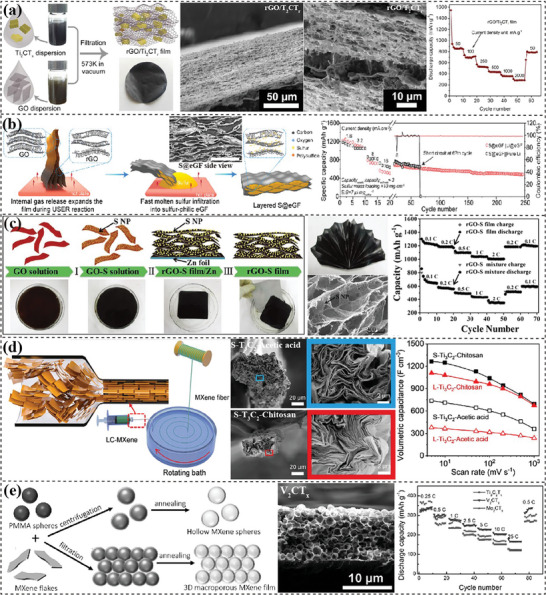
Templated pores designed in 2D materials‐based flexible electrodes. a) Gas‐shaped porous structure in filtered rGO/Ti_2_CT_r_. Reproduced with permission.^[^ ^234^ ^]^ Copyright 2018, John Wiley and Sons. b) Expanded rGO film absorbing molten S for high‐performance Li‐S batteries. Reproduced with permission.^[^ ^237^ ^]^ Copyright 2020, Elsevier. c) Zn‐induced self‐assembly of rGO/S hydrogel film. Reproduced with permission.^[^ ^242^ ^]^ Copyright 2016, John Wiley and Sons. d) Porous MXene fibers spun from LC MXene dispersion from a fast solvent‐exchanging process. Reproduced with permission.^[^ ^146^ ^]^ Copyright 2020, American Chemical Society. e) Porous MXene film shaped by poly(methyl methacrylate) (PMMA) spheres. Reproduced with permission.^[^ ^247^ ^]^ Copyright 2017, John Wiley and Sons.

***Gases*** generated from the decomposition of surface groups on 2D nanosheets or the introduced materials expand the stacked layers forming porous structures.^[^ ^231^, ^232^, ^233^ ^]^ It is evident that the removal of oxygen‐containing groups on GO or MXene surface generates gas species. Han and coworkers^[^ ^234^ ^]^ expanded the tightly packed GO/Ti_2_CT_x_ layers by utilizing the gases generated from a thermal reduction treatment (**Figure** **8**a). The formed film displayed a much more open layer structure with interconnected channels, which displayed a high surface area of 270 m^2^ g^−1^ and a high capacity of 325 mAh g^−1^ at 2 A g^−1^. By introducing hydrazine vapor as reducing agent, Niu et al.^[^ ^235^ ^]^ demonstrated that the rapidly evolved gas species during the reduction process were conducive to forming highly porous rGO film, and the amount of hydrazine was used to control the porosity. Cui's group^[^ ^236^, ^237^, ^238^ ^]^ developed an ultrafast self‐expansion and reduction (USER) process to prepare porous rGO films for hosting lithium or sulfur. Upon contacting a superheated medium, the fast decomposition of abundant functional groups on GO nanosheets generated abundant gas, which forced compact rGO layers to expand (Figure 8b). The expanded rGO films (eGF) with nanoscale gaps were then adopted to absorb molten lithium or sulfur for producing high‐performance electrodes. The strong binding with lithium and a low Li nucleation barrier of eGF realized a low overpotential and a flat voltage profile.^[^ ^236^ ^]^ The high tortuosity and S‐philicity of eGF effectively restrict the shuttle effect of sulfur.^[^ ^237^ ^]^ The good mechanical properties of eGF also ensured a low dimension variation during cycling and good flexibility. As a result, the full battery with Li‐eGF anode and S@eGF cathode demonstrated a stable cycling performance.

***Liquid droplets*** are a kind of soft templates used to form pores in 2D materials‐based flexible electrodes. The large aspect ratio and impermeability of 2D nanosheets facilitate the entrapment of solvent molecules (e.g. water droplets) between stacked layers during wet‐processing treatment. Sublimation of crystalline water (i.e. a freeze‐drying process) prevents the collapsing and restacking of 2D nanosheets forming stable porous structures.^[^ ^239^, ^240^ ^]^ Porous polypyrrole‐mediated graphene foam obtained by freeze‐drying reached an extra‐high specific surface area of 463 m^2^ g^−1^.^[^ ^241^ ^]^ Niu's group^[^ ^242^ ^]^ utilized metal‐induced self‐assembly process to fabricate S‐decorated rGO nanosheets on Zn substrate (Figure 8c). After freeze‐drying, rGO/S aerogel acquired a tensile strength of 68 MPa, a high surface area of 635 m^2^ g^−1^, a high capacity of 1252 mAh g^−1^ at 0.1 C, and a capacity retention of 77% at 2 C. A solvent‐exchanging process during wet‐spinning also entraps solvent droplets between 2D nanosheets. Aboutalebi et al.^[^ ^145^ ^]^ demonstrated that fast water extraction from an acetone coagulation bath increased the amount of entrapped water and formed a highly porous structure in the wet‐spun LCGO fiber, achieving a boosted surface area of 2210 m^2^ g^−1^. Similar phenomenon was also observed for wet‐spun MXene fibers (Figure 8d):^[^ ^146^ ^]^ much denser and conductive MXene fibers were formed from slow coagulation in a chitosan bath which displayed the highest volumetric capacitance of 1265 F cm^−3^, while higher porosity was derived from a fast coagulation in acetic acid bath which achieved a higher gravimetric capacitance of 434 F g^−1^.

***Solid templates*** are typically sacrificial nanoparticles^[^ ^243^, ^244^ ^]^ or supporting skeletons^[^ ^245^ ^]^ in porogen leaching methods. They provide solid support and their removal forms controllable pores.^[^ ^243^, ^244^ ^]^ Zhao et al.^[^ ^246^ ^]^ employed sulfur particles as sacrificial templates to construct porous Ti_3_C_2_T_x_ film through filtration and sublimation. The Ti_3_C_2_T_x_ film with the highest sulfur content of 71% exhibited the smallest density of 0.25 g cm^−3^ and highest BET surface area of 39.9 m^2^ g^−1^. As a result, it delivered the best performance of 314.9 mAh g^−1^ after 300 cycles at 0.05 A g^−1^, and 220 mAh g^−1^ at 1 A g^−1^ after 3500 cycles in LIBs. Polymer‐based nanoparticles are another commonly used solid templates due to their high processability and easy removal.^[^ ^244^ ^]^ Gogotsi's group^[^ ^247^ ^]^ applied poly(methyl methacrylate) (PMMA) spheres as templates to prepare porous MXene films through a filtration and post‐annealing treatment (Figure 8e). Among them, the 3D V_2_CT_x_ film displayed the highest capacity of 310 mAh g^−1^ after 1000 cycles at 0.5 A g^−1^ in a sodium‐ion battery^[^ ^247^ ^]^ while the porous Ti_3_C_2_T_x_ film possessed excellent pseudocapacitive behavior with an ultra‐high rate capability of 210 F g^−1^ at 10 V s^−1[^ ^248^ ^]^ and a high capacity of 210 mAh g^−1^ at 0.05 A g^−1^ in a Mg‐ion battery.^[^ ^249^ ^]^

To guide the growth of 2D materials to form 3D porous structures, metal frameworks are typically applied as sacrificial templates.^[^ ^250^, ^251^ ^]^ Nickel foam has been extensively studied to prepare light and conductive graphene foams (GFs) which reached a surface area of up to 850 m^2^ g^−1^ via chemical vapor deposition.^[^ ^252^, ^253^, ^254^ ^]^ GFs can be further adopted to load other active materials (e.g. MoS_2_) by taking advantage of their highly conductive and porous structure, maximizing electrolyte accessibility for improved ion diffusion kinetic through the dual paths for ion diffusion.^[^ ^118^, ^119^, ^120^ ^]^

### 3.3 Atomic‐Scale Design Strategies

Atomic‐scale design strategies directly adjust the crystal structure of 2D materials to tune ion permeability, electron transport and activities. Atoms in 2D nanosheets can be removed to generate in‐plane holes,^[^ ^255^ ^]^ substituted (i.e. heteroatom doping)^[^ ^256^ ^]^ or rearranged (i.e. phase engineering).^[^ ^257^ ^]^ The application of atomic‐scale design strategies endows 2D nanosheets with more exposed active sites, higher conductivity, larger interlayer distance, and improved electrochemical activity.^[^ ^258^ ^]^

#### 3.3.1 Holey Structures

The impermeability of 2D nanosheets decreases ion diffusivity and results in unsatisfactory electrochemical performance. Ion‐permeable holes from the selective etching of atoms on 2D nanosheets provide an effective solution to enhance ion diffusion along the direction perpendicular to the planes of 2D materials.^[^ ^259^ ^]^ Moreover, the holey structure also increases surface area and creates defects for high ion storage.^[^ ^260^, ^261^ ^]^ Holey structures have been demonstrated for graphene,^[^ ^262^, ^263^, ^264^ ^]^, MXenes^[^ ^265^ ^]^ and MoS_2_.^[^ ^266^, ^267^ ^]^ However, no holey MoS_2_‐based flexible electrodes has been reported yet.

**Figure 9:**
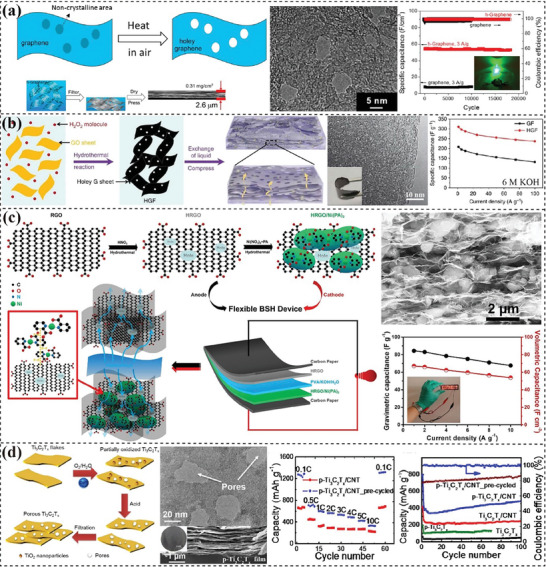
Holey structured 2D nanosheets. a) Thermally prepared holey graphene for high performance supercapacitors. Reproduced with permission.^[^ ^263^ ^]^ Copyright 2014, American Chemical Society. b) Holey graphene produced through a H_2_O_2_ involved chemical activation process. Reproduced with permission.^[^ ^272^ ^]^ Copyright 2014, Springer Nature. c) Flexible battery‐supercapacitor hybrid device assembled with HRGO/Ni(picolinic acid)_2_ cathode and HRGO anode. Reproduced with permission.^[^ ^278^ ^]^ Copyright 2021, Elsevier. d) Holey MXene (p‐Ti_3_C_2_T_x_) film and p‐Ti_3_C_2_T_x_/CNT film for high lithium storage. Reproduced with permission.^[^ ^265^ ^]^ Copyright 2016, John Wiley and Sons.

***Holey graphene*** can be obtained through template‐etching^[^ ^268^, ^269^ ^]^, template‐growing,^[^ ^270^ ^]^ thermal oxidation^[^ ^262^, ^263^, ^264^ ^]^ and wet‐chemical etching^[^ ^271^, ^272^, ^273^ ^]^. The templated methods can generate controllable and ordered pores but with low production efficiency, unsuitable for practical applications. Thermal oxidation and wet‐chemical etching offer more effective ways to design and optimize holey structures. Thermal oxidation is applied to oxidize partial carbon atoms on graphene planes under heating conditions. Lin et al.^[^ ^262^ ^]^ first deposited Ag nanoparticles on graphene nanosheets to catalyze the oxidation process. The obtained holey graphene displayed a BET surface area in the range of 280 to 380 m^2^ g^−1^. They revealed that the nonocrystalline spots on graphene sheets directly reacted with oxygen in hot air (**Figure** **9**a).^[^ ^263^ ^]^ The formed pores not only elevated the BET surface area from 471 m^2^ g^−1^ to 658 m^2^ g^−1^, but also improved the volume density of the filtered holey graphene film to 1.2 g cm^−3^. As a result, the holey graphene film delivered a comparable gravimetric capacitance of 45 F g^−1^ to graphene (40 F g^−1^), but with much higher volumetric performance of 53 F cm^−3^ (12 Wh L^−1^) vs. 8 F cm^−3^ (2 Wh L^−1^) at 3 A g^−1^. In addition, a prolonged air oxidation process (430 °C/10 h) was beneficial for formation of mesopores and simultaneous introduction of oxygen atoms, contributing to a greatly enhanced capacitance of 72 F g^−1^ at 10 A g^−1^ compared with only 33 F g^−1^ from a 3h treatment.^[^ ^264^ ^]^

Wet chemical etching is also used to fabricate holey graphene sheets. Chemicals such as KOH,^[^ ^274^ ^]^ HNO_3_,^[^ ^255^ ^]^ H_2_O_2_,^[^ ^272^, ^273^ ^]^ H_2_SO_4_,^[^ ^260^ ^]^ and NaI^[^ ^275^ ^]^ can react with the active carbon atoms around the defective sites on the basal planes creating uniform in‐plane nanopores. Holey graphene activated by KOH could reach a high surface area of 3 100 m^2^ g^−1^, even surpassing the theoretical value of 2 630 m^2^ g^−1^ for pristine graphene.^[^ ^274^ ^]^ Duan's group^[^ ^272^ ^]^ developed a H_2_O_2_‐assisted hydrothermal etching method to create active defective sites on the basal plane of GO (Figure 9b). Abundant in‐plane nanopores on graphene sheets increased the BET surface area to 830 m^2^ g^−1^, much higher than 260 m^2^ g^−1^ for non‐holey graphene aerogel. As expected, the compressed flexible HG film electrodes exhibited an ultrahigh capacitance of 310 F g^−1^ at 1 A g^−1^ and 237 F g^−1^ at 100 A g^−1^. By manipulating the processing parameters, they successfully produced solution dispersible holey graphene oxide (HGO) with excellent processability.^[^ ^276^ ^]^ Flexible films prepared by filtering reduced HGO delivered a capacitance of 201 F g^−1^ at 1 A g^−1^ and 140 F g^−1^ at 20 A g^−1^ in solid‐state supercapacitors. Furthermore, HGO nanosheets were robust for loading other active materials.^[^ ^277^ ^]^ In Yang's work,^[^ ^278^ ^]^ Ni(picolinic acid)_2_ loaded holey reduced graphene oxide (HRGO/Ni(PA)_2_) was selected as cathode coupled with HRGO anode to assembly a flexible battery‐supercapacitor hybrid (BSH) device (Figure 9c), which achieved a high capacitance of 67.1 F cm^−3^ at 1 A g^−1^ (0.79 A cm^−3^) with a retention rate of 83.3% after 10 000 cycles at 10 A g^−1^.

***Holey MXene*** was first reported by Gogotsi's group^[^ ^265^ ^]^ in 2016. It was synthesized by employing metal cations (Cu^2+^, Co^2+^ or Fe^2+^) to catalyze the oxidation sites of MXene with oxygen in water (Figure 9d). Uniform pores in tens of nanometers dimensions were created on MXene sheets resulting in increased surface area and pore volume of 93.6 cm^2^ g^−1^ and 0.25 cm^3^ g^−1^ compared with only 19.6 cm^2^ g^−1^ and 0.023 cm^3^ g^−1^ for pristine Ti_3_C_2_T_x_. The filtered freestanding and flexible holey Ti_3_C_2_T_x_ film exhibited superior lithium storage performance of 110 mAh g^−1^ after 100 cycles at 0.5 C. The porous Ti_3_C_2_T_x_/CNT composite electrode delivered a high capacity of ≈1250 mAh g^−1^ at 0.1 C and a remarkable rate performance. Recently, their group proposed a H_2_SO_4_‐assisted etching way to prepare holey MXene layers.^[^ ^279^ ^]^ The increased ion pathways along the direction perpendicular to MXene nanosheets contributed to an excellent rate performance, a 64% capacitance retention (208 F g^−1^/756 F cm^−3^) as scan rates increased from 5 to 10 000 mV s^−1^. Chen and coworkers^[^ ^280^ ^]^ used large amounts of gases generated from pyrolysis of urea to create macro‐pores on MXene nanosheets. The formed holey MXene electrode exhibited a high performance improvement to 203 F g^−1^ at 5 A g^−1^ from 82 F g^−1^ for pristine MXene.

#### 3.3.2 Heteroatom‐Doping Structures

Heteroatom doping is a method to introduce foreign atoms into the lattice structure of 2D materials for adjusting electronic structure.^[^ ^281^ ^]^ Introduction of atom dopants induces distorted structures and regulates electronic band of host materials, which lead to the modulation of physicochemical characteristics including surface area,^[^ ^282^, ^283^ ^]^ electrical conductivity,^[^ ^284^, ^285^ ^]^ interlayer spacing^[^ ^286^ ^]^ and electrochemical activity.^[^ ^287^ ^]^

**Figure 10:**
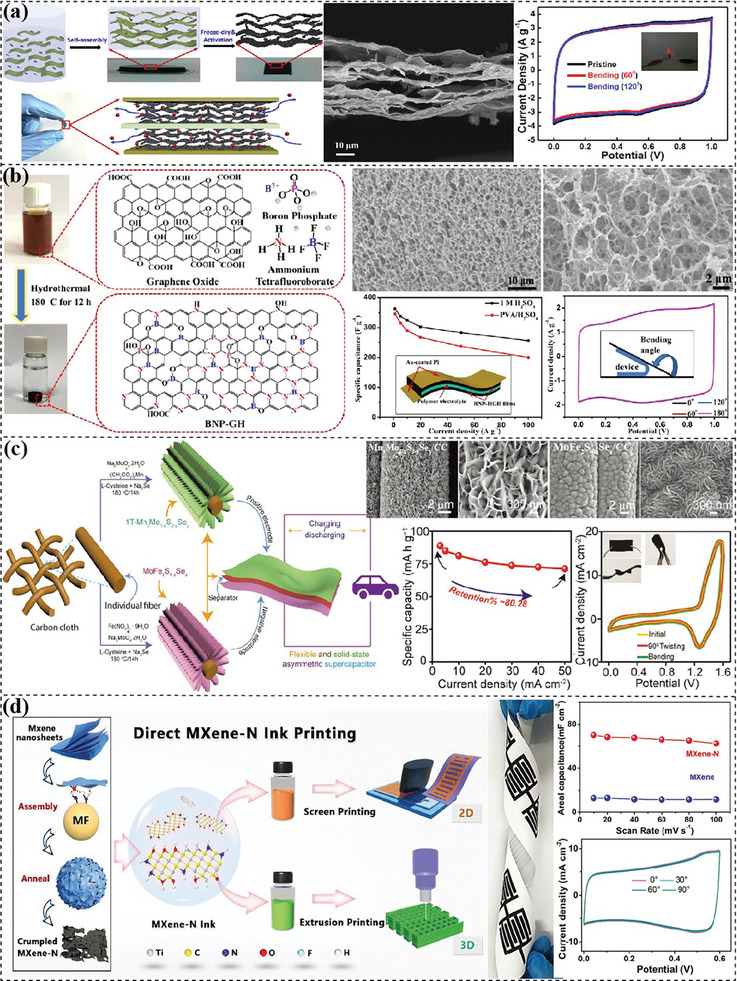
Flexible electrodes prepared from heteroatoms‐doped 2D materials. a) Porous P‐doped graphene films for high‐performance ASSCs. Reproduced with permission.^[^ ^283^ ^]^ Copyright 2018, Elsevier. b) A ternary‐doped (B, N, and P) holey graphene hydrogel for high mass‐loading ASSCs. Reproduced with permission.^[^ ^256^ ^]^ Copyright 2018, Elsevier. c) 1T‐Mn_x_Mo_1–x_S_2–y_Se_y_ and MoFe_2_S_4–z_Se_z_ grown on carbon cloths (CC) for asymmetric ASSCs. Reproduced with permission.^[^ ^305^ ^]^ Copyright 2020, John Wiley and Sons. d) N‐doped MXene ink for printing flexible microsupercapacitors devices.^[^ ^316^ ^]^ Copyright 2019, John Wiley and Sons.

***Heteroatom‐doped graphene***. Graphene is the first engineered 2D material with heteroatom‐doping strategy. The typical doped heteroatoms are non‐metal elements including nitrogen,^[^ ^288^, ^289^ ^]^ boron,^[^ ^290^, ^291^ ^]^, sulfur,^[^ ^285^, ^292^ ^]^ phosphorus^[^ ^283^ ^]^ and fluorine.^[^ ^293^ ^]^ Liu and co‐workers^[^ ^282^ ^]^ demonstrated that nitrogen doping improved the BET surface area to 431 m^2^ g^−1^, achieving a high capacitance of 3.4 mF cm^−2^ at 20 mA cm^−2^ in inkjet‐printed N‐doped graphene MSCs. Ajayan's group^[^ ^294^ ^]^ deposited a N‐doped graphene film on Cu foil which delivered almost doubled capacity than pristine graphene in LIBs, due to the enriched surface defects and introduced pyridinic N atoms. Peng et al.^[^ ^295^ ^]^ reported laser processing of polyimide/H_3_BO_3_ sheet to form B‐doped graphene. A three times higher areal capacitance (16.5 mF cm^−2^) was obtained compared with the undoped sample, owing to faster ion transport and better electrode‐electrolyte interface. In Subramaniyam's work,^[^ ^285^ ^]^ sulfur doping was confirmed to enable a high conductivity of 114 S cm^−1^ as well as a large surface area for better contact with the electrolyte. Flexible S‐doped graphene electrode displayed unprecedented electrochemical performance in a symmetric aqueous supercapacitor (367 F g^−1^ at 1 A g^−1^), Li‐ion battery (1697 mA h g^−1^ at 100 mA g^−1^), and Na‐ion battery (472 mA h g^−1^ at 50 mA g^−1^). Yu et al.^[^ ^283^ ^]^ used phytic acid as a phosphorus precursor to prepare a hierarchically porous P‐doped graphene (HPG) film through sonication‐mixing, vacuum filtration, freeze‐drying and thermal annealing (**Figure** **10**a). As electrodes in ASSCs, the capacitance was increased to as high as 144 F g^−1^ from 84 F g^−1^ for undoped HG. The in situ FT‐IR and ex situ XPS analysis illustrated that the redox activity of P≐O sites of C─P≐O bonding in P‐doped graphene accounted for the excellent pseudocapacitive behavior. Additionally, different heteroatoms were introduced jointly to harvest the synergistic effects for fascinating properties and performance.^[^ ^296^, ^297^, ^298^ ^]^ The redox sites generated by co‐doped oxygen and nitrogen atoms in rGO foam contributed to a high pseudocapacitance proportion of 65%.^[^ ^299^ ^]^ Pan et al.^[^ ^256^ ^]^ developed ternary‐doped (B, N, and P) holey graphene and processed it into a hierarchical hydrogel (BNP‐GH) with an extra‐high surface area of 980 m^2^ g^−1^ (Figure 10b). Density functional theory (DFT) calculations revealed its extra‐high adsorption energy with H_2_SO_4_ electrolyte (229.5 kJ mol^−1^). By combining those benefits, BNP‐GH flexible electrodes with a commercial‐level mass loading of 10 mg cm^−2^ delivered a high capacitance of 316 F g^−1^ at 1 A g^−1^ in ASSCs.

***Heteroatom‐doped TMDs***. MoS_2_ is the main studied TMD material in this field. The reported dopants include metals (Co, Sb, Cu, Ni, Fe, Mn, etc.) and non‐metals (P, C, N, O, Se, etc.).^[^ ^42^, ^228^, ^300^, ^301^, ^302^, ^303^, ^304^, ^305^, ^306^ ^]^ For example, Co‐doped MoS_2_ are able to release structure stress and transform semiconducting 2H MoS_2_ to metallic 1T MoS_2_ with increased conductivity.^[^ ^303^ ^]^ First‐principles calculations revealed the role of Co doping in reducing volume deformation for excellent cycling capability.^[^ ^300^ ^]^ The introduction of phosphorus atoms into MoS_2_ not only increased the surface area from 41.2 to 62.9 m^2^ g^−1^ and electrical conductivity from 4.6 × 10^−4^ to 2.9 × 10^−3^ S cm^−1^, but also enlarged the interlayer distance to 0.679 nm with an improved Na^+^ diffusion coefficient from 10^−6^ to 10^−4^ cm^2^ s^−1^.^[^ ^304^ ^]^ However, the low aspect ratio, easy‐agglomeration and lack of functional groups make it hard to obtain binder‐free MoS_2_ electrodes.^[^ ^25^ ^]^ Lee's group^[^ ^305^ ^]^ employed carbon cloth (CC) as substrate to synthesize hierarchical 1T‐Mn_x_Mo_1–x_S_2–y_Se_y_ (Mn, Se co‐doped MoS_2_) and MoFe_2_S_4–z_Se_z_ (Fe, Se co‐doped MoS_2_) through a simple hydrothermal reaction (Figure 10c). The Mn_x_Mo_1–x_S_2–y_Se_y_/CC sample possessed a surface area of 71 m^2^ g^−1^ and an areal capacity of 0.76 mAh cm^−2^ (288 mAh g^−1^) at 1 mA cm^−2^, higher than 48.5 m^2^ g^−1^ and 0.534 mAh cm^−2^ (218 mAh g^−1^) for MoFe_2_S_4–z_Se_z_/CC. In the flexible asymmetric ASSCs, a large potential window of 0 −1.6 V was achieved with a high capacity of 91 mAh g^−1^ at 3 mA cm^−2^, 73 mA h g^−1^ at 50 mA cm^−2^ and a retention ratio of 83.5% after 10, 000 cycles at 25 mA cm^−2^.

***Heteroatom‐doped MXenes***. Both metal and non‐metal dopants, such as Zn, V, Nb, P, B, N, O and S, are used to tune the properties of MXenes.^[^ ^8^, ^307^, ^308^, ^309^, ^310^, ^311^ ^]^ The incorporation of Nb in MXene (Ti_3_C_2_) decreased the bandgap for higher conductivity,^[^ ^312^ ^]^ while the sulfur^[^ ^313^ ^]^ and nitrogen^[^ ^314^ ^]^ doping effectively increased the interlayer distance for high diffusivity. Yang et al.^[^ ^315^ ^]^ demonstrated that the optimized N doping increased the capacitance. Yu et al.^[^ ^316^ ^]^ employed a melamine formaldehyde templating method to prepare crumpled N‐doped MXene (MXene‐N), which was processed into inks for screen‐printing of planar pattern electrodes and extrusion‐printing of fiber‐stacked 3D electrodes (Figure 10d). Benefitting from the 3D crumpled structure with fast electrolyte transport channels and abundant nitrogen active sites, MXene‐N displayed a greatly improved performance. Under different bending angles, no obvious performance drop was detected. In Yu's work,^[^ ^317^ ^]^ a hydrothermal process with excess cysteine was developed to control the oxidation of MXene while simultaneously doping N atoms. The flexible film electrodes made of N‐doped holey MXene nanosheets with surface‐decorated TiO_2_ nanoparticles gave a high capacitance of 426.5 mF cm^−2^ at 0.5 mA cm^−2^, 373.12 F cm^−2^ at 2.5 mA cm^−2^ and a capacitance retention of 74.89% after 10 000 cycles at 2 mA cm^−2^ in PVA/LiCl ASSCs. Multi‐element doping that involves the introduction of several different heteroatoms can result in large lattice distortion to improve ion diffusion and create more active sites for achieving high electrochemical performance. This method has been adopted to engineer MXene,^[^ ^318^, ^319^ ^]^ but is still at the early stage of development for flexible electrodes.

#### 3.3.3 Phase Engineering

**Figure 11:**
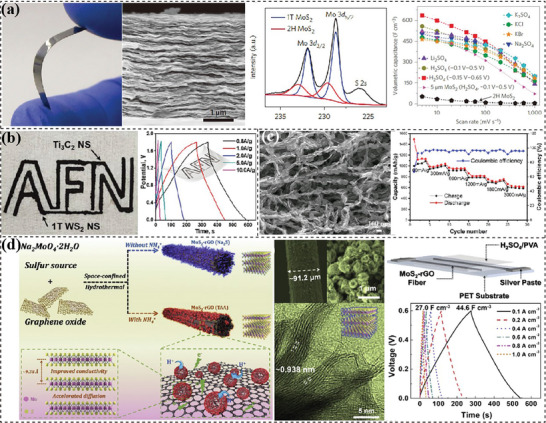
Phase engineering in 2D materials‐based flexible electrodes. a) Restacked 1T‐rich MoS_2_ films for high‐performance SCs. Reproduced with permission.^[^ ^320^ ^]^ Copyright 2015, Nature. b) Exfoliated 1T‐phase WS_2_ for fabricating asymmetric supercapacitors with Ti_3_C_2_ electrode. Reproduced with permission.^[^ ^326^ ^]^ Copyright 2020, John Wiley and Sons. c) Growth of 1T‐MoSe_2_ on CNTs to prepare hybridized flexible film electrodes with high lithium storage performance. Reproduced with permission.^[^ ^329^ ^]^ Copyright 2017, American Chemical Society. d) Hydrothermally synthesized 1T‐enriched MoS_2_‐rGO hybrid fibers for high‐performance fibrous supercapacitors. Reproduced with permission.^[^ ^257^ ^]^ Copyright 2020, Elsevier.

As is well known, 1T phase TMDs can achieve metallic properties (10−100 S cm^−1^) to satisfy the demand for fast electron transport and high electrochemical kinetics in EES devices.^[^ ^320^ ^]^ Compared to semiconductive 2H phase, 1T structure also possesses the advantage of higher binding energy with various electrolyte ions and polysulfides.^[^ ^8^, ^321^ ^]^ Phase engineering strategies have been developed to fabricate stable 1T‐TMDs. The organolithium‐assisted exfoliation process of MoS_2_ is accompanied by the insertion of Li ions into MoS_2_, which induces the phase transfer from semi‐conductive 2H to metallic 1T phase.^[^ ^322^, ^323^, ^324^, ^325^ ^]^ Chhowalla et al.^[^ ^320^ ^]^ reported the fabrication of Li‐intercalated exfoliated MoS_2_ nanosheets and the corresponding filtered films with a high 1T metallic phase of 70% for supercapacitors (**Figure** **11**a). In a three‐electrode system, it displayed a high capacitance of ≈400 to ≈700 F cm^−3^ at 20 mV s^−1^ in a variety of aqueous electrolytes (H^+^, Li^+^, Na^+^, and K^+^). 1T‐WS_2_ exfoliated by a similar method was used to prepare in‐plane asymmetric supercapacitors with Ti_3_C_2_ as counter electrode and polyester/cellulose blend (PCB) cloth as substrate (Figure 11b).^[^ ^326^ ^]^ The flexible devices demonstrated excellent cycling stability, which can be integrated with a force sensor in a lightweight and wearable bio‐monitoring system.

The simple and widely used hydrothermal process is also explored to produce TMDs with high content of 1T phase for flexible electrodes.^[^ ^117^, ^327^, ^328^ ^]^ In Xiang's work,^[^ ^329^ ^]^ a one‐step solvothermal method was used to deposit hierarchical MoSe_2_ nanosheets on activated single‐wall carbon nanotubes (SWCNT) film (Figure 11c). The oxygen‐containing groups on SWCNTs benefited the formation of Mo─O─C bonding for the growth of vertical MoSe_2_ nanosheets with good structural stability, while the insertion of N,N‐dimethylfumarate (DMF) solvent into MoSe_2_ layers resulted in an enlarged interlayer distance of 1 nm and stabilized metallic 1T phase. Hybridized structure and electrical coupling between 1T‐MoSe_2_ and SWCNTs ensured a high‐speed electron/ion transfer in the flexible and binder‐free anodes for superior lithium storage: 971 mAh g^−1^ after 100 cycles at 0.3 A g^−1^ and 630 mAh g^−1^ at 3 A g^−1^. Yu and co‐workers^[^ ^257^ ^]^ showed that using thioacetamide (TAA) as a sulfur precursor in the microfluidic‐spinning approach simultaneously sulfurized MoO_4_^2−^ into MoS_2_ and released NH_4_^+^ to intercalate into the MoS_2_ interlayers for a larger interlayer spacing of 0.938 nm (Figure 11d). With rGO as supporting material, the obtained MoS_2_‐rGO hybrid fibers possessed a higher content of 1T crystal phase than the control sample using Na_2_S as a sulfur source. As a result, the capacitance of MoS_2_‐rGO (TAA) was enhanced to 313.2 F cm^−3^ (at 2 A cm^−3^) in ASSCs from only 134 F cm^−3^ for MoS_2_‐rGO (Na_2_S).

## 4 Conclusion and Perspective

**Figure 12:**
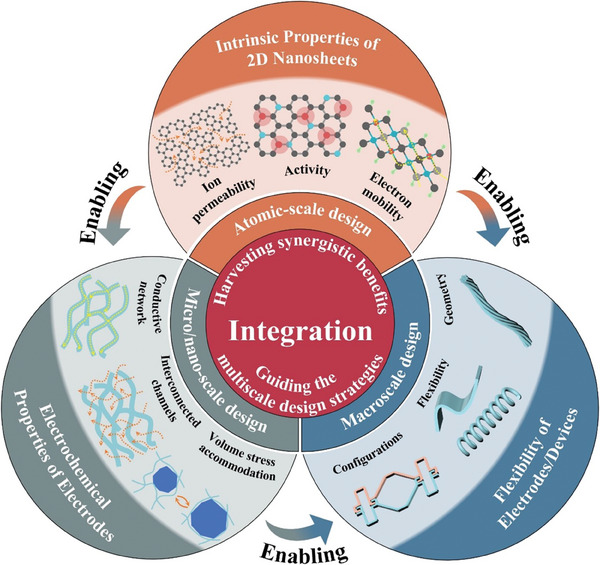
The underlying mechanisms of multiscale structural designs in 2D materials‐based flexible electrodes and the provision of guidelines for future development.

Wearable electronics and corresponding flexible energy storage devices have become increasingly important due to their ability to seamlessly integrate technology into our everyday wearables. These devices offer personalized, portable, and unobtrusive solutions for tracking health, communication, accessing information, and performing tasks hands‐free. Flexible energy storage devices address the critical need of power supplies for wearable electronics, providing lightweight, flexible, and compatible energy storage solutions. The promising development in this field is driven by advancements in designing electrolyte, electrode, and the integrated devices. 2D nanomaterials, especially graphene, TMDs and MXenes, have demonstrated tremendous potential in the field of flexible energy storage electrodes and devices, owing to their inherent 2D structure and attractive physicochemical and electrochemical properties. Considerable efforts have been devoted to developing multiscale design strategies to overcome the drawbacks of 2D materials (such as restacking, low vertical diffusivity and volume expansion) and realize high‐performance flexible electrode. Macroscale strategies focus on designing the shapes of electrodes and devices to improve mechanical stability and deformability while delivering high electrochemical performance. Micro/nano‐scale strategies adjust the morphologies and micro‐/nano‐ structures of electrode materials to enhance electrochemical reaction kinetics and structural robustness, while atomic‐scale strategies directly tailor the intrinsic structures of 2D nanosheets. They are all crucial elements in determining the performance of flexible electrodes. The integration of these complementary strategies endows electrodes with excellent electrochemical performance and mechanical deformability (**Figure** **12**).

At macroscale, the applied strategies focus on shaping the geometry of flexible electrodes including 2D membranes, 1D fibers and in‐plane interdigital patterns, in order to improve the flexibility and electrochemical performance. The corresponding device is in sandwich, parallel/twisted/concentric and in‐plane interdigitated configurations, respectively. These strategies have demonstrated the effectiveness in realizing flexible electrodes and devices with high performance and high deformability. For example, kirigami patterns^[^ ^189^ ^]^ are applied to construct interconnected device arrays with high‐degree of stretchability, while fibrous devices are suitable for integration with clothes. To promote practical applications, the simplifying or optimizing of the fabrication process to make it compatible with existing fabrication approaches is highly desirable. For instance, the roll‐pressing technique that can prepare dry and flexible electrodes has good adaptability with the common roll‐to‐roll production line. However, currently the application of this technique is hindered by the non‐uniform distribution of binders and the poor strength and adhesion with substrates, which calls for the development of new binders.^[^ ^130^ ^]^ Moreover, the widely used liquid electrolyte in flexible devices inevitably brings leakage and safety issues. Gel‐state electrolyte and solid electrolyte are preferred due to their high strength, good stability and high safety. However, they suffer from low ion diffusivity and poor interfacial contact with electrode materials especially under deforming conditions. On this basis, ensuring the compatibility and adhesion between electrode and electrolyte becomes particularly crucial for realizing safe flexible devices. It necessitates the careful design of interfacial structures through surface modifications, interlayer coatings, or introduction of interface‐enhancing materials.^[^ ^330^ ^]^

The rational design strategies at micro‐/nano‐scales facilitate fast electron/ion transport across 2D materials‐based flexible electrodes and accommodation of volume stress upon cycling. The constructed architectures can be mainly classified into stacked layers, nanofiber meshes and templated porous structures. A highly porous structure with superhigh surface area has been certified as an effective solution to improve ion diffusivity for high‐rate capacity as well as address the volume change problem for long cycle life.^[^ ^331^, ^332^ ^]^ However, it is usually accompanied by limited volumetric energy density and deteriorated flexibility. There exists a trade‐off in‐between. It is necessary to comprehensively evaluate the influence of 3D porous structures on the overall performance to identify the optimal design. Applying advanced micro‐/nano‐structures such as 2D confinement structures^[^ ^39^, ^333^ ^]^ may be a promising research direction for developing high‐performance 2D materials‐based flexible electrodes. 2D confinement structures can well utilize the confinement effect of subnanometric stacked nanosheets to obtain both enhanced ion diffusivity and dense structures for good rate capability and high energy density. However, the strong restacking tendency of 2D nanosheets makes it difficult to construct 2D confinement structures, especially on a large scale for flexible electrodes. The subnanometric stacking kinetics of 2D nanosheets and the influence of introduced materials on the stacking kinetic need to be revealed, in order to facilitate the engineering of controllable spacings for constructing large‐range 2D confinement structure.

At atomic scales, the created in‐plane holes, heteroatom‐doping and phase engineering alter the electronic structure of 2D materials thus affecting ion permeability, electron mobility, and chemical/electrochemical activity. The intrinsic properties can be controllably adjusted via these strategies which lay the foundation for designing macroscale and micro‐/nano‐scale structures. Further exploration of their potential in 2D materials‐based flexible electrodes is highly demanded and will have a profound influence on the construction of micro/nano and macro‐scale structures. Atomic‐scale structures are the basic units for constructing interfacial structures between 2D materials and other active materials, and the achieving of fast interfacial electron transfer and good structural stability require both experimental and theoretical research. For instance, holey MoS_2_ nanosheets are not strong enough to fabricate freestanding flexible electrode by themselves. The introduction of high‐strength materials (e.g. graphene, carbon nanotubes, MXenes) forming composites are expected to well utilize the unique characteristics of holey MoS_2_ for achieving fascinating performance. Moreover, the structure‐property relationship induced from atomic‐scale design strategies have not been fully understood, owing to the complex interaction between various doped atoms/introduced defects and the difficulty to conduct atomic‐scale experimental investigation. The increasingly attractive multi‐element doping strategy has been adopted to engineer 2D materials, in which the complex interactions between different heteroatoms and their effects on the properties are not conclusive yet.^[^ ^256^, ^318^ ^]^ Calculations based on density functional theory (DFT) can accelerate the research in this direction by analyzing the total energy and electronic structures with corroboration from experimental results.^[^ ^191^, ^334^ ^]^

Overall speaking, atomic‐scale strategies focus on regulating the intrinsic properties of 2D nanosheets including active sites, ion permeability through nanosheets, ion diffusion along nanosheets, and electron transport within nanosheets; micro‐/nano‐scale strategies play a crucial role in addressing the challenges associated with the skeletons of flexible electrodes which require low interfacial resistance, high diffusion rate and accommodation to volume stress; macroscale strategies focus on the electrode shapes and deformability issues as well as the fabrication techniques. These factors make up a complex system in flexible electrodes determining electrochemical performance and mechanical properties. Despite significant progress has been achieved in the advancement of high‐performance 2D materials‐based flexible electrodes, there are still numerous obstacles to overcome in the collaborative design of multiscale structures. The subsequent discussion presents the potential avenues for enhancing multiscale structures, along with suggested solutions and perspectives.

- The structural evolution of 2D nanomaterials during electrochemical processes has not been completely revealed yet, especially for those undergoing huge volume change or conversion reactions. By applying high‐tech methods, a deep understanding of the dynamic change of structures and the relationship with electrode performance can be achieved to further guide the design of multiscale structures. In‐situ techniques are powerful in investigating the structural evolution. For instance, in‐situ spectroscopic techniques (e.g. XRD, Raman) characterize the evolution of electronic and crystal structures, while in‐situ electron microscopy (e.g. TEM) observes the dynamics of micro‐/nano‐structures.
- The reported structure‐property relationship of 2D nanomaterials‐based flexible electrodes usually focus on individual structure, while the dependencies and interactions between different structures are hardly discussed despite they influence the performance from different aspects. For example, stacked nanosheets with horizontal channels are conducive to in‐plane patterned devices, but not suitable for sandwich‐configurated devices owing to its low vertical diffusion rate.^[^ ^335^ ^]^ The combination of holey nanosheets and 3D porous structure achieve high surface and rapid horizontal and vertical diffusion, but with decreased mechanical strength.^[^ ^272^ ^]^ Therefore, the complicated interactions between multiscale structures require meticulous design for their integration complementing the benefits.
- Very recently, AI (artificial intelligence) have demonstrated great potential to providing valuable insights and patterns in the materials field through deep learning and systematically analyzing large‐scale datasets.^[^ ^336^ ^]^ They can be employed to assist multiscale design by identifying optimal structural configurations and simulating their synergistic effects. Application of advanced research tools are anticipated to accelerate the development of multiscale designing strategies for guiding the preparation of 2D materials‐based flexible electrodes.

In conclusion, designing multiscale structures is effective to solve the problems and acquire high performance flexible electrodes based on 2D nanomaterials and beyond, producing high‐performance flexible energy storage devices to meet the requirement for next‐generation wearable electronics.

## Conflict of Interest

The authors declare no conflict of interest.
